# Role of Dermoscopy in the Assessment of Basal Cell Carcinoma

**DOI:** 10.3389/fmed.2021.718855

**Published:** 2021-08-20

**Authors:** Loredana Ungureanu, Ioana Cosgarea, Simona Şenilǎ, Alina Vasilovici

**Affiliations:** ^1^Department of Dermatology, Iuliu Haţieganu University of Medicine and Pharmacy, Cluj-Napoca, Romania; ^2^Department of Dermatology, Translational and Clinical Research Institute, Newcastle University, Newcastle upon Tyne, United Kingdom

**Keywords:** basal cell carcinoma, dermoscopy, diagnosis, treatment, excision margins

## Abstract

Basal cell carcinoma is one of the most common cancers in white people, with a continuous increase worldwide. Dermoscopy, a non-invasive technique, allows early diagnosis based on the presence of typical vascular structures, pigmented structures, and ulceration and the absence of specific melanocytic structures. Moreover, dermoscopy is useful in basal cell carcinoma management, enabling the differentiation between multiple histological subtypes, between pigmented and non-pigmented variants and allowing a more accurate assessment of surgical margins. After non-ablative therapies, dermoscopy allows the accurate detection of residual disease. The purpose of this review is to highlight the dermoscopic features encountered in basal cell carcinoma and to outline the role of dermoscopy for diagnosis and therapeutic response in this cancer.

## Introduction

Basal cell carcinoma (BCC) is one of the most common cancers in white people, with an incidence that increases continuously worldwide (1). Mortality is low, but morbidity is high, and the burden on healthcare services is significant (1). Metastases in BCC are extremely rare, and reliable risk factors for systemic dissemination are unknown. Therefore, therapy is primarily influenced by the risk of subclinical infiltration and local recurrence (2). Treatment guidelines divide BCC into low-risk and high-risk tumors, based on patient, clinical and pathological characteristics (2). Various histopathologic subtypes of BCC respond significantly different to surgical and non-surgical treatment options (3). While imiquimod and PDT are first line options for superficial BCC (sBCC), surgical excision is the treatment of choice for nodular BCC (nBCC). Mohs' surgery is considered gold standard in the most aggressive subtypes of BCC (infiltrative and morpheiform) due to the high rate of recurrence after standard surgery (3).

Studies show that dermoscopy, a non-invasive technique for the diagnosis of skin lesions, improves the diagnostic accuracy of BCC (4–8). Recent evidence suggests that dermoscopy is also useful in BCC management, providing information on the histological subtype, the presence of pigmentation or ulceration, as well as the response rate to non-ablative therapies (3).

The aim of the present review is to describe the role of dermoscopy in the assessment of basal cell carcinoma, from diagnosis to therapeutic response.

## Dermoscopy for the Diagnosis of Basal Cell Carcinoma

Dermoscopic diagnosis in BCC is based mainly on the presence of typical vascular structures, pigmented structures, ulceration, and the absence of specific melanocytic structures. In a recent meta-analysis, Reiter et al. showed that dermoscopy is a sensitive and specific tool for the diagnosis of BCC and should complete clinical examination of a suspected skin lesion, especially in pigmented tumors (9). Dermoscopy improved the diagnostic sensitivity from 66.9 to 85% (*p* = 0.0001) and the specificity from 97.2 to 98.2% (*p* = 0.006) compared with naked eye examination alone (9).

### Vascular Dermoscopic Structures

**Figure 1:**
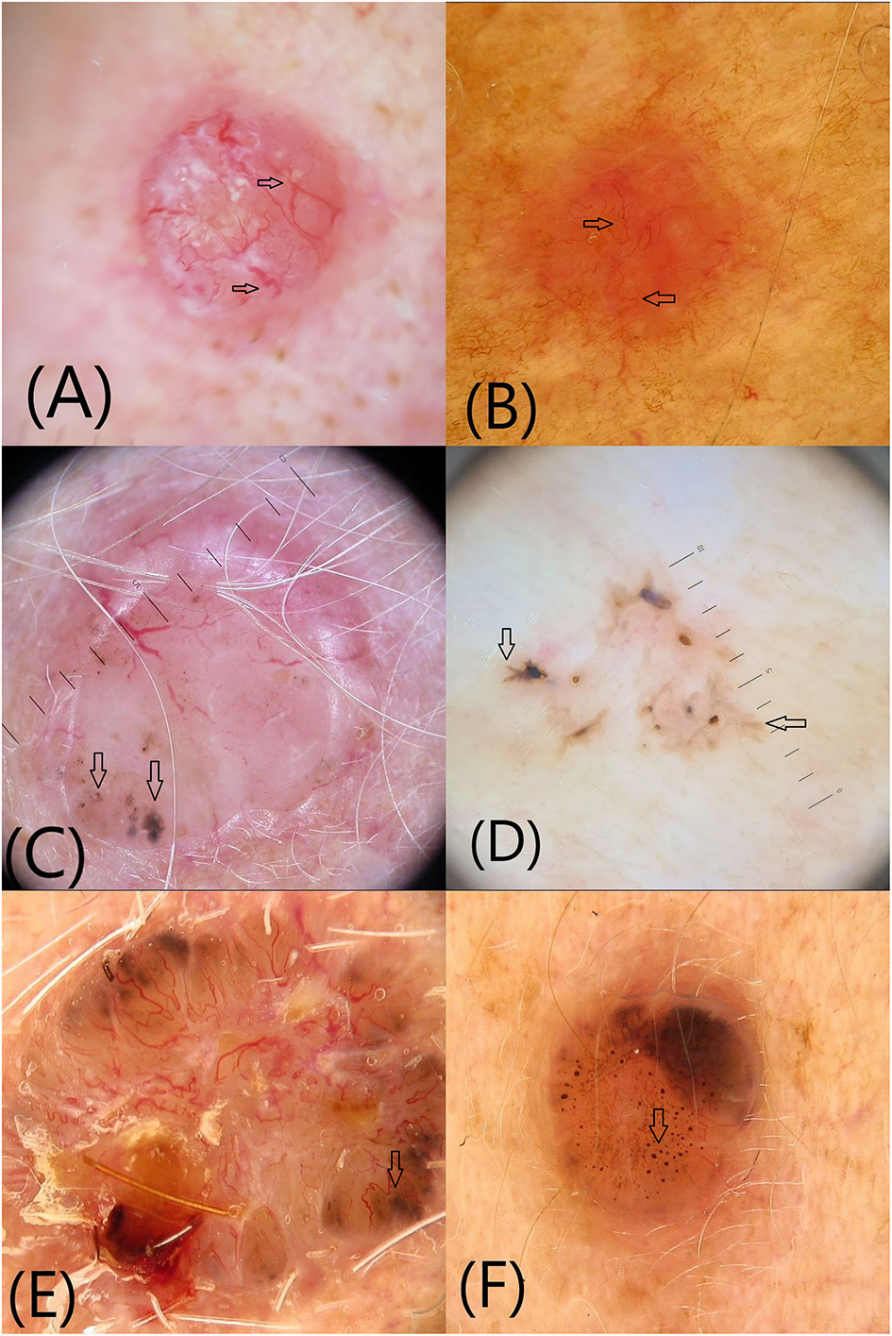
Vascular and pigmented dermoscopic structures (arrows): **(A)** Arborising vessels; **(B)** Short fine telangiectasias **(C)** Blue-gray globules and dots **(D)** Maple leaf-like areas **(E)** Spoke-wheel structures **(F)** In-focus dots.

*Arborizing vessels* are the first vascular structures that have been described in BCC (Figure 1A). They are bright red, usually no longer than 1 mm, large diameter vessels, branching irregularly into finer capillaries. Due to their superficial location just beneath the epidermis they are clearly visible, being described as in focus (3, 4). Arborizing vessels were described overall in 60.7% of all BCC, being more frequent in nBCC compared with sBCC (10). Histopathologically, arborizing vessels correspond to dilated vessels in the dermis (3).

The second most common vascular pattern observed in BCC is *short fine telangectasias* (Figure 1B) (3, 4, 10). They are described as vessels with a small diameter and length of <1 mm, with few or no branches. Histopathologically, they correspond to telangectatic vessels located in the papillary dermis (3, 4, 10). Short fine telangectasias are significantly more frequent in sBCC compared with nBCC (10).

Occasionally, BCC may show vascular structures that are typically seen in other types of tumors, such as hairpin, glomerular, dotted and comma vessels (10). A polymorphous pattern is characteristic for amelanotic/hypomelanotic melanoma and squamous cell carcinoma, being rarely observed in BCC (10).

### Pigmented Dermoscopic Structures

The largest pigmented structures observed in BCC are *blue-gray ovoid nests*, described as well-circumscribed, confluent or near confluent pigmented ovoid or elongated structures, larger than globules and not intimately connected to the pigmented tumor body (3, 4, 11). Histologically, they correspond to well-defined, large tumor nests with pigment aggregates, invading the dermis (3, 4, 11). Blue-gray ovoid nests are described in all subtypes of BCC except of sBCC (3).

*Blue-gray globules and dots* are smaller than nests and appear as well-circumscribed, pigmented, loosely arranged round or oval structures (Figure 1C). They correlate histopatologically to small tumor nest in the papillary and/or reticular dermis (11). Blue-gray globules and dots can be seen in all types of BCC. A particular type of dots described in BCC are *in-focus dots*, a term used to describe loosely arranged well-defined small gray dots, which appear sharply in focus, reflecting free pigment deposits along the dermo-epidermal junction and/or melanophages in the papillary or reticular dermis (Figure 1F) (3).

*Maple leaf-like areas* are bulbous extensions connected to a common base, producing a leaf-like pattern at the periphery of the tumor that never arises from pigmented network or from adjacent confluent pigmented areas (Figure 1D). Histopathologically, they correspond to multifocal tumor nests containing pigment aggregates, connected to each other by lobular extensions, localized in the epidermis and papillary dermis. Maple leaf-like areas are highly specific for BCC, especially the superficial subtype (3, 4, 11).

*Spoke-wheel structures/areas* consist of well-circumscribed radial projections connected to a more strongly pigmented central axis (Figure 1E). The projections are usually tan but sometimes blue or gray, while the central axis is dark brown, black or blue. They correspond to tumor nests connected to the epidermis, characterized by finger-like projections and centrally located pigmentation. Spoke-wheel structures are highly specific for BCC, especially the superficial subtype (3, 11).

*Concentric structures* are thought to be variations of spoke wheel areas seen more frequently in the superficial subtype of BCC. They are defined as irregularly shaped globular-like structures with different colors and a darker central area (3, 11).

Heavily pigmented BCCs may present dermoscopic features associated with melanocytic lesions, such as brown globules, peppering or a blue-white veil, making the differential diagnosis difficult (11, 12).

### Ulceration/Multiple Small Erosions

**Figure 2:**
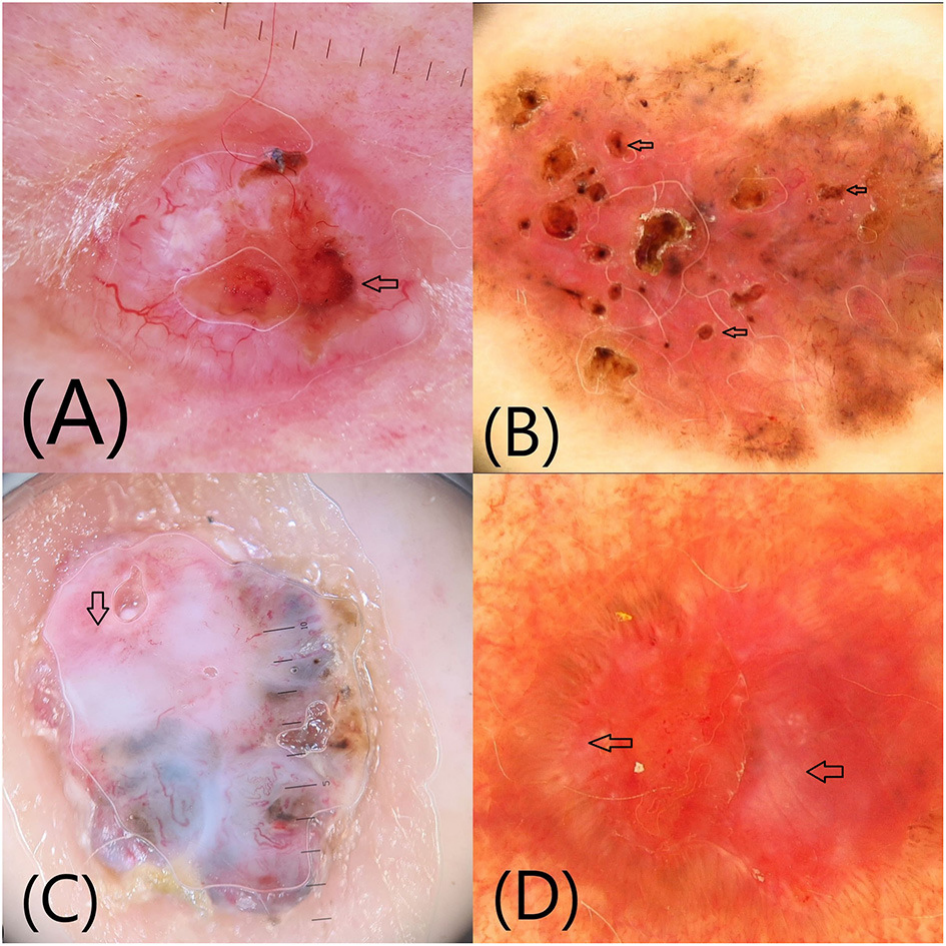
Additional dermoscopic structures (arrows): **(A)** Ulceration **(B)** Multiple small erosions **(C)** Shiny white-red structureless areas **(D)** Shiny white lines.

*Ulceration* is the loss of the epidermis and a portion of the dermis, usually covered by haematogenous or serous crusts (Figure 2A). By dermoscopy, ulceration may be seen as one or more large structureless areas of orange-red to black-red color. Ulceration appears typically in nBCC (3, 4, 11).

*Multiple small erosions* are smaller in size than ulcerations and histopathologically correspond to superficial losses of the epidermis covered by thin crusts (Figure 2B). They are seen as small, reddish-brown or yellow-orange structureless areas, and are typically described in sBCC (3, 4, 11).

### Other Dermoscopic Structures

*Shiny white-red structureless areas* have been described especially in sBCC and correspond to diffuse dermal fibrosis or fibrotic tumor stroma (Figure 2C) (11). *Shiny white lines* can only be visualized by polarized light as orthogonal short and thick crossing lines (Figure 2D). Histopathologically they correspond to the presence of collagenous stroma and fibrosis in the dermis (3, 4, 11). The combination of shiny white-red structureless areas and shiny white lines is highly suggestive for BCC (11).

Pyne et al. recently described a *circumferential stellate pattern* extending from the visible margin of the tumor in infiltrative BCC. This may be formed by white lines, vessels, or skin surface folds (13). Imbernón-Moya et al. described non-pigmented bulbous structures comparable to maple leaf-like areas, named negative maple leaf-like structures that interrupt structures in the surrounding skin (telangiectasias, solar lentigines) and help delineate non-pigmented BCC (14).

Navarrete-Dechent et al. described multiple aggregated yellow-white globules, defined as multiple, aggregated, white-to-yellowish globules arranged in clusters, visible in both polarized and non-polarized light, positively associated with histologic high-risk BCC subtypes. Histopathologically, they were correlated with dystrophic calcifications, which are more common in histologic high-risk subtypes (15). Multiple aggregated yellow-white globules are different from shiny white structures that do not form globules and are only seen under polarized light (15).

## Dermoscopy for Predicting the Histological Subtype

According to the risk of recurrence, BCCs can be divided into (1) histologic low risk subtypes: nodular, superficial, infundibulocystic, fibroepithelial, and (2) histologic high risk subtypes: basosquamous carcinoma, sclerosing/morphoeic, infiltrating, BCC with sarcomatoid differentiation, micronodular (16). The response rates of different subtypes to surgical and non-surgical treatment methods vary significantly. While non-surgical treatments, such as imiquimod and photodynamic therapy are recommended as first-line options for the management of sBCC, surgical excision remains the treatment of choice for other subtypes, including nodular, micronodular, morphoeic, and infiltrating BCC. Mohs surgery is the golden standard for high-risk recurrent BCCs, especially in critical anatomic areas, because it offers the highest cure rates (1, 16). As a consequence, the distinction between low-risk and high risk subtypes has important therapeutic implications.

It has been shown that dermoscopic structures correlate to morphologic criteria observed in histopathology. As a consequence, different histologic subtypes display different dermoscopic patterns (3, 4, 6, 8, 11, 17).

### Superficial Basal Cell Carcinoma

Multiple small erosions, superficial fine telangiectasias and shiny white or red structureless areas characterize sBCC. However, the most specific features are observed in pigmented sBCC and are represented by spoke-wheel and maple leaf-like structures (3, 4, 11, 17).

### Nodular Basal Cell Carcinoma

nBCC is characterized by the presence of arborizing vessels, large blue-gray ovoid nests, multiple blue-gray dots, globules and ulceration, arborizing vessels being the most typical feature (3, 4, 11). The concomitant presence of arborizing vessels and ulceration suggests a BCC with a high risk of local recurrence (18).

### Morpheaform (Sclerodermiform) Basal Cell Carcinoma

Conforti et al. defined the dermoscopic criteria associated independently with mBCC as compared to other subtypes (19). They showed that ulceration was significantly more frequent in mBCC, followed by fine arborizing vessels, pink-white areas and multiple blue-gray dots and globules (19). Linear branched vessels in mBCC are usually finer, more scattered and with fewer ramifications than the arborizing vessels of nBCC (3).

### Infiltrative Basal Cell Carcinoma

Pampena et al. described the clinical and dermoscopic criteria that can differentiate infiltrative BCC from other histologic subtypes (20). The authors showed that infiltrative BCC frequently displays ulceration and a mix of arborizing and superficial fine telangiectasia. Shiny white structures, such as short white streaks and red-white structureless areas, were also frequently seen (20). The most important difference between infiltrative and nodular BCC upon dermoscopy is the higher occurrence of superficial fine telangiectasia in the former. Telangiectasia of iBCC have smaller caliber and less tendency to branch compared with those of nBCC. In iBCC, however, arborizing vessels and superficial fine vessels can coexist in the same lesion (20). A “stellate pattern” described as “vessels, white lines or skin folds radiating from the peripheral margin of the tumor into the surrounding skin” was associated with iBCC (13).

As previously mentioned, multiple aggregated yellow-white globules, defined as multiple, clustered, white-to-yellowish globules have been in high-risk BCC histologic subtypes such as mBCC and iBCC (15).

### Fibroepithelioma of Pinkus

Fibroepithelioma of Pinkus is characterized by fine arborizing vessels in the center and dotted vessels at the periphery on a white-pinkish background (3). White streaks, brown-gray structureless areas and blue-gray dots can also be seen (21). Recently, a negative pigment network or white network was described in such lesions (22).

### Basosquamous Carcinoma

In BSC dermoscopy reveals simultaneously features related to BCC and features related to squamous cell carcinoma. Giacomel at al. found that the most common dermoscopic structures were unfocused peripheral arborizing vessels, superficial scales, keratin masses, ulceration or blood crusts, white structures, and blue-gray blotches (23). Akay et al. found that keratin masses were the most common dermoscopic structures seen in BSC along with arborizing vessels (24). Other features included surface scaling, ulceration, white structureless areas, white clods, blood spots (24). The authors concluded that BSC is characterized by a vascular pattern suggestive of BCC associated with dermoscopic features of keratinization (24).

### Micronodular Basal Cell Carcinoma

The presence of truncated vessels and multiple blue-gray globules has been reported in micronodular BCC (18, 25).

## Dermoscopy for Basal Cell Carcinoma Management

### Dermoscopy for Choosing the Apropriate Therapy

Studies show that dermoscopy is important not only in BCC diagnosis, but also for choosing the best management strategy. The optimal treatment choice depends on several factors, prediction of the histopathological subtype being one of the most important ones. In regard to therapy, the most important distinction that has to be made is between superficial and non-superficial BCC. Lallas et al. showed that the presence of maple leaf-like areas, multiple small erosions and short thin telangiectasias together with the absence of blue-gray ovoid nests, arborizing vessels, and ulceration is highly predictive for sBCC, with a sensitivity of 81.9% and a specificity of 81.8% (17).

The presence of pigment is another factor that influences the response to therapy. It has been shown that pigment reduces significantly the response rate of the tumor to photodynamic therapy (3). Pigmentation can be present in any of the previous histologic subtypes of BCC. Although, pigmentation is most frequently evident on clinical examination, a recent study showed that 30% of clinically non-pigmented BCCs displayed dermoscopic pigmentation (26). In sBCC and iBCC pigment is located at the dermoepidermal junction and appears brown in color, as maple-leaf-like areas, spoke-wheel areas, concentric structures and in-focus dots. In nBCC pigment is located in the deeper dermis and appears blue or gray in color as blue–gray ovoid nests and blue–gray globules (4).

Urech at al. showed that dermoscopy can be used as an indicator for treatment response to imiquimod. The authors showed that dermoscopic detection of erosions or ulceration is a strong predictor of favorable response to imiquimod (27).

### Dermoscopy for Evaluation of Response to Non-ablative Therapies

Non-ablative therapeutic modalities are frequently used for the management of sBCC with high response rates. However, it is difficult to assess whether the lesion has been completely eradicated or if residual disease is still present (28). Apalla et al. showed that dermoscopy is helpful in the assessment of treatment outcome and monitoring of sBCC. They showed that residual disease-associated dermoscopic criteria (pigmented structures, ulceration, and arborizing telangiectasias) are associated with tumor persistence, while the disappearance of dermoscopic criteria of sBCC correlates with complete clearance. Moreover, reappearance of any such dermoscopic criteria during follow-up should raise suspicion of tumor recurrence (27). The authors underlined that close monitoring is mandatory in order to recognize early recurrence in case of post-treatment presence of white or red structureless areas and/or superficial fine telangiectasias (28).

### Dermoscopy for Margin Detection in Surgical Excision

Incomplete and suboptimal surgical excision is an important factor affecting recurrence rates. Carducci et al. showed that preoperative digital dermoscopy is a better method than clinical evaluation to detect presurgical excision margins in nBCC of the head and neck (29). In a following study, the authors succeeded to extend the observations, demonstrating that greater pre-surgical improvement is obtained by dermoscopy in iBCC and mBCC as well (30).

## Conclusions

Dermoscopy, a non-invasive diagnostic technique, improves sensitivity and specificity in basal cell carcinoma diagnosis. Moreover, dermoscopy is a useful tool that enables the distinction between different histological subtypes of BCC, helping the clinician to choose the best therapeutic strategy. Following non-ablative treatment, dermoscopy allows an accurate assessment of the therapeutic response, while when used before surgical excision, it aids in the accurate detection of tumoral margins, improving surgical performance.

## Author Contributions

SS and IC: collected the literature. LU and AV: literature review, manuscript drafting, and critical revision of the manuscript for important intellectual content. All authors contributed to manuscript revision, read, and approved the submitted version.

## Conflict of Interest

The authors declare that the research was conducted in the absence of any commercial or financial relationships that could be construed as a potential conflict of interest.

## Publisher's Note

All claims expressed in this article are solely those of the authors and do not necessarily represent those of their affiliated organizations, or those of the publisher, the editors and the reviewers. Any product that may be evaluated in this article, or claim that may be made by its manufacturer, is not guaranteed or endorsed by the publisher.
